# Osteopontin genetic variants are associated with overall survival in advanced non-small-cell lung cancer patients and bone metastasis

**DOI:** 10.1186/1756-9966-32-45

**Published:** 2013-07-24

**Authors:** Yunzhen Chen, Haichun Liu, Wenliang Wu, Yi Li, Jianmin Li

**Affiliations:** 1 Department of Orthopedic, Qilu Hospital of Shandong University, No.107, Wen Hua Xi Road, Jinan 250012, Shandong province, China

**Keywords:** Osteopontin, Non-small-cell lung cancer, Genetic variants, Bone metastasis

## Abstract

**Purpose:**

Osteopontin (OPN) plays important roles in the modulation of apoptosis, angiogenesis, immune response, and tumor invasion. Elevated osteopontin expression has been reported in the lung cancer tissues compared to counterpart normal tissues. This study examined whether genetic variations in the osteopontin gene are associated with survival of lung cancer patients and occurrence rate of bone metastasis.

**Experimental design:**

Three hundred and sixty patients with stages I to IV between 2003 and 2007 were recruited in this study and same number of healthy persons were used as control. Three promoter osteopontin polymorphisms, OPN-66 T/G, -156G/GG, and -443C/T variants were genotyped using DNA from blood lymphocytes. Chi-square test and a Fisher’s exact test were used to analyze the genotype distribution among TNM stages and incidence of bone metastasis and lymph mode metastasis. Kaplan-Meier method and log-rank test were used to compare survival by different genotypes.

**Results:**

For the variant at nt −443 (CC), there was a significant difference between the number of patients with stage IV and those with all other stages of lung cancer (p < 0.01). Patients with −443 (CC) variant had significant higher incidence of bone metastasis development compared to other genotypes. For the variant at nt −443 (CT), there was a significant difference between the number of lung cancer patients with stage III + IV and those with stage I + II (P < 0.01). The survival rates for patients with the C/C genotype were significantly lower than for patients with the other two genotypes (C/T, T/T).

**Conclusion:**

OSTEOPONTIN −443C/T polymorphism is a potential predictive marker of survival in lung cancer patients, it is correlated with bone metastasis significantly.

## Introduction

Lung cancer is the most common malignancy all over the world and the leading cause of death in men [1], and non-small cell lung cancer (NSCLC) accounts for >80% of primary lung cancers [2,3]. Treatment of these patients is usually based on a multidisciplinary strategy, including a combination of radiotherapy and chemotherapy. However, results of these treatments were unsatisfactory with a 3-year overall survival (OS) being 10% to 20% [4]. The classic prognostic determinants for lung cancer include the tumor-node-metastasis staging system, performance status, sex, and weight loss. Unfortunately, all these factors are far less than sufficient to explain the patient-to-patient variability. Therefore, identification of new biomarkers for more accurate prognostic and predictive assessment is warranted and could be helpful to highlight the possibility of patient-tailored decisions [5].

The skeleton is the most common site for distant metastasis in patients with cancer [6]. Tumor cells homing to form bone metastases is common in non-small cell lung cancer (NSCLC), just like what is seen in breast, prostate and thyroid cancers [7,8]. Some patients may experience bone metastasis many years after surgery of the primary tumor. The high morbidity and significantly increased risk of fractures associated with bone metastasis seriously affect patients’ quality of life. About 36% of all lung cancers and and 54.5% of stage II-IIIA NSCLC showed postoperative recurrence or metastasis [9]. Many lung cancer patients expect new and more sensitive markers to predict metastatic diseases. If bone metastasis can be predicted early enough, then effective prevention could be started and may result in an improvement in survival [10]. The molecular and cellular mechanisms leading to the development of bone metastasis in NSCLC remain unclear, so searching for effective biomarkers to predict the possibility of bone metastasis is valuable in clinical practice.

OPN is a sibling glycoprotein that was first identified in 1986 in osteoblasts. OPN is a highly negatively charged, extracellular matrix protein that lacks an extensive secondary structure [11]. The OPN gene is composed of 7 exons, 6 of which contain coding sequence [12]. OPN was first implicated in malignancy by in vitro studies detecting increased levels of OPN expression after cell transformation [13] and from the observation that tumor cells with high metastatic potential had increased OPN expression. As discussed, OPN binds to several integrin receptors including α4β1, α9β1, and α9β4 expressed by leukocytes. These receptors have been well-established to function in cell adhesion, migration, and survival in these cells. Therefore, recent research efforts have focused on the role of OPN in mediating such responses [14]. OPN gene transcription in bone tissue is regulated by the interaction between transactivating factors and vitamin D3 responsive elements [15].

Previous study has confirmed that OPN is overexpressed in the NSCLC tumor tissues compared to adjacent normal counterparts; and its overexpression is significantly correlated with TNM stages and lymph metastasis [16]. However there are no relative reports about the relationship between OPN polymorphisms with survival of NSCLC and risk of bone metastasis currently. In the present study, we recruited 360 NSCLC patients and 360 cancer-free control, aim to investigate whether OPN-66 T/G, -156G/GG, and -443C/T genotypes affect the survival of patients; meanwhile to determine whether they have an association with incidence of bone metastasis development.

## Patients and methods

### Patients

Three hundred sixty ambulatory patients with stage I to IV lung cancer patients who were admitted to the College of Medicine of Shan Dong University, Qi Lu Hospital in Jinan, China between October 2003 and July 2007 were studied. 79 patients with bone metastasis and 281 patients without bone metastasis were included in this study. The median age was 57.21 years (range, 24 to 81 years); 199 patients were male and 161 patients were female. The diagnosis of lung cancer was confirmed cytologically or histologically. All patients gave their informed consent to the diagnostic procedures. The TNM stage mentioned in the current study was diagnosed at first hospitalization. Healthy control group consisted of a random sample of 360 ethnic Han Chinese from Shan Dong province.

Bone metastasis evaluation: All patients were evaluated for bone metastasis by bone scintigraphy. A total of 25 mCi 99mTechnetium methylene diphosphonates (MDP) was injected intravenously, and front and back images of the whole body were taken after 3 hours. The apparatus used was a double-detector gamma camera (VERTEX, ADAC Co., CA, USA). Bone scintigraphy was read by two radiologists and classified into either a bone metastasis-positive or a negative group. When the bone scintigraphic interpretation differed among radiologists, positive scans were further assessed by additional radiographs; computerized tomography, magnetic resonance imaging, positron emission tomography or bone biopsy, except when the increased uptake was recognized as being due to a benign condition [17,18].

All the participants agreed to participate in this study and had adequate blood DNA for genotyping and all had complete follow-up and clinical information. There was no significant difference in the distribution of demographic information between patients enrolled and patients who did not. Written informed consent was obtained from each participant for the use of their DNA and clinical information. The study was approved by the ethnic committee of Qilu hospital.

### SNP genotyping

Genomic DNA was extracted from 5-mL blood sample that was collected from each patient upon recruitment. The OPN-66 T/G, -156(rs17524488), and −443(rs11730582) variants were genotyped by direct sequencing of the sense and anti-sense strands following polymerase chain reaction (PCR) amplification of the promoter regulatory region −473 to −3 (forward primer 50-CAA GCT ACT GCA TAC TCG AAA TCA CA-30; reverse primer 50- ACA ACC AAG CCC TCC CAG AAT TTA-30), as previously described [19]. PCR was performed using 50 ng DNA as a template under the following conditions: 95°C for 10 min, then 36 cycles of 94°C for 30 s, an annealing temperature for 60 s, and 72°C for 60 s, with a final extension at 72°C for 15 min. After affinity membrane purification using the QIAquick Gel Extraction kit (Qiagen, Carlsbad, CA, USA), the PCR products were subjected to cycle sequencing with the respective forward and reverse primer using an automated ABI 3100 DNA sequencer by GeneCore Bio Technologies (Shanghai China). A 15% blind, random sample of study subjects was genotyped twice by different persons (Yunzhen Chen and Haichun Liu) and the reproducibility was 100%.

### Statistical analysis

Statistical analysis was performed using SPSS 18.0 software. Quantitative variables departing from the normal distribution, including age, gender and smoking status were summarized. Comparison of age between cases and controls was assessed using an independent Student’s t-test. Comparison of gender, smoking stauts and genotype frequencies between cases and controls was assessed using a chi-square test and a Fisher’s exact test. Survival was calculated by the Kaplan-Meier method. All probability (P) values were two-tailed and statistical significance was indicated as P < 0.05.

## Results

### Patient characteristics and clinical outcomes

This study recruited 360 patients with lung cancer and 360 healthy controls. The baseline clinical characteristics of patients are summarized in Table 1. 79 patients (17.4%) were diagnosed with bone metastasis. By the time of the final analysis (July 2012), the median follow-up time had been 32 months. There were no significant differences in terms of distribution of age and gender, but significant on smoking status, suggest smoking is one of risk factors. Clinicopathologic characteristics of the patients and controls are shown in Table 1.

**Table 1:** Clinicopathologic characteristics of patients with NSCLC and healthy controls

	**No. of patients or controls**		
**Characteristics**	**Case (n)**	**Controls (n)**	**P**
No.	360	360	
Age, y			>0.05
Median	57.2	56.3	
Range	24-81	23-87	
Gender			>0.05
Male	199	197	
Female	161	163	
Smoking status			<0.01
Never	210	258	
Former	56	43	
Current	94	59	
KPS		-	-
≥80	289	-	
<80	71	-	
Histology		-	
Squamous carcinoma	213	-	
Adenocarcinoma	111	-	
Others	36	-	
Tumor stage at diagnosis		-	
I	81	-	
II	96	-	
III	82	-	
IV	101	--	
Lymph node		--	
Positive	223	-	
Negative	137	-	
Bone metastasis		-	
Yes	79	-	
No	281	-	

Note: For the smoking status, classification for tobacco consumption is never (<100 cigarettes lifetime), former (> 100 cigarettes lifetime and quit >12 months prior) and current.

### SNPs in the promoter region of human OPN gene

Direct sequencing of DNA fragments between nt −473 and nt −3 in patients and age- and gender-matched controls revealed 3 SNPs in the OPN promoter, located at nt −156 [GG/GG homozygotes, GG/G-(deletion) heterozygotes, G-/G- homozygotes], nt −443 [CC homozygotes, CT heterozygotes, TT homozygotes], and nt −66 (Additional file 1: Figure S1), as shown in Table 2. There was no significant difference in the distribution of these SNPs (nt −66, -156, -443) between patients and controls. The distribution of genotypes for TNM stages in lung cancer is shown in Table 3. However, regarding tumor-node-metastasis TNM stages, we found that for the SNP at nt −443, among patients with the CT genotype, there was a significant difference between patients with stages I + II and stages III + IV (p < 0.01), data was shown in Table 4. Similarly, among patients with the CC genotype at nt −443, there was a significant difference between patients with stages III + IV and stages I + II (P < 0.01) and between stages IV and combination of stage I to stage III (P < 0.01; Table 4). There were no significant differences among the TNM stages and the other two SNPs (nt −66 and nt −156) of the OPN promoter. We also found that significant association between the −443 genotypes in the OPN promoter and lymph node metastasis, type CC and CT had more risks to develop lymph node metastasis (Table 2).

**Table 2:** Comparison of OPN promoter between lung cancer patients and healthy controls

	**Controls**	**Patients**		**Lung cancer**					
	**n**	**n**	**P**	**LN(+)**	**LN(−)**	**P**	**BM(+)**	**BM(−)**	**P**
−66 T/G									
TT	351	356	1.00	221	135	1.00	77	279	1.00
TG	9	4	0.262	2	2	0.637	2	2	0.211
−156									
G/G	155	137	1.00	83	54	1.00	26	96	1.00
G/GG	136	150	0.094	89	61	0.391	39	126	0.671
GG/GG	69	73	0.218	48	25	0.550	14	59	0.855
−443									
TT	153	164	1	49	115	1.00	23	141	1.00
CT	163	165	0.388	93	72	<0.001	36	129	0.084
CC	44	31	0.068	25	6	<0.001	20	11	<0.001

Note: *LN* Lymph node metastasis, *BM* bone metastasis. P value was calculated by chi-square test and a Fisher’s exact test.

**Table 3:** The distribution of genotypes for TNM stages among lung cancer patients

	**The TNMs of lung cancer**				
**Genotypes**	**I**	**II**	**III**	**IV**	**P**
−66					0.624
TT	81	94	81	100	
TG	0	2	1	1	
−156					0.711
G/G	35	41	40	39	
G/GG	31	36	31	38	
GG/GG	15	19	11	24	
−443					<0.001
TT	48	51	26	39	
CT	31	41	51	42	
CC	2	4	5	20	

Note: P value refers to significance value among all the groups on SNP, and was calculated by person chi-square test.

**Table 4:** The genotype distribution of nt −443 in the OPN promoter by lung cancer TNM stage

	**The TNM stages of lung cancer**					
**Genotypes**	**I + II**	**III + IV**	**P**	**I + II + III**	**IV**	**P**
−443						
TT	99	65	1.000	125	39	1.000
CT	72	93	0.003	123	42	0.798
CC	6	25	<0.001	11	20	<0.001

### Effect of SNPs on bone metastasis

As shown in Table 2, there were total 31 patients who had CC genotype at nt −443, among them, 20 cases were at stage IV. Surprisingly, all of these 20 cases were diagnosed with bone metastasis. By compared with TT genotype, it demonstrated that CC genotype at nt-443 might significantly increase the risk of development of bone metastasis (p < 0.01).

### Associations between genotypes in the OPN promoter region and survival

**Figure 1:**
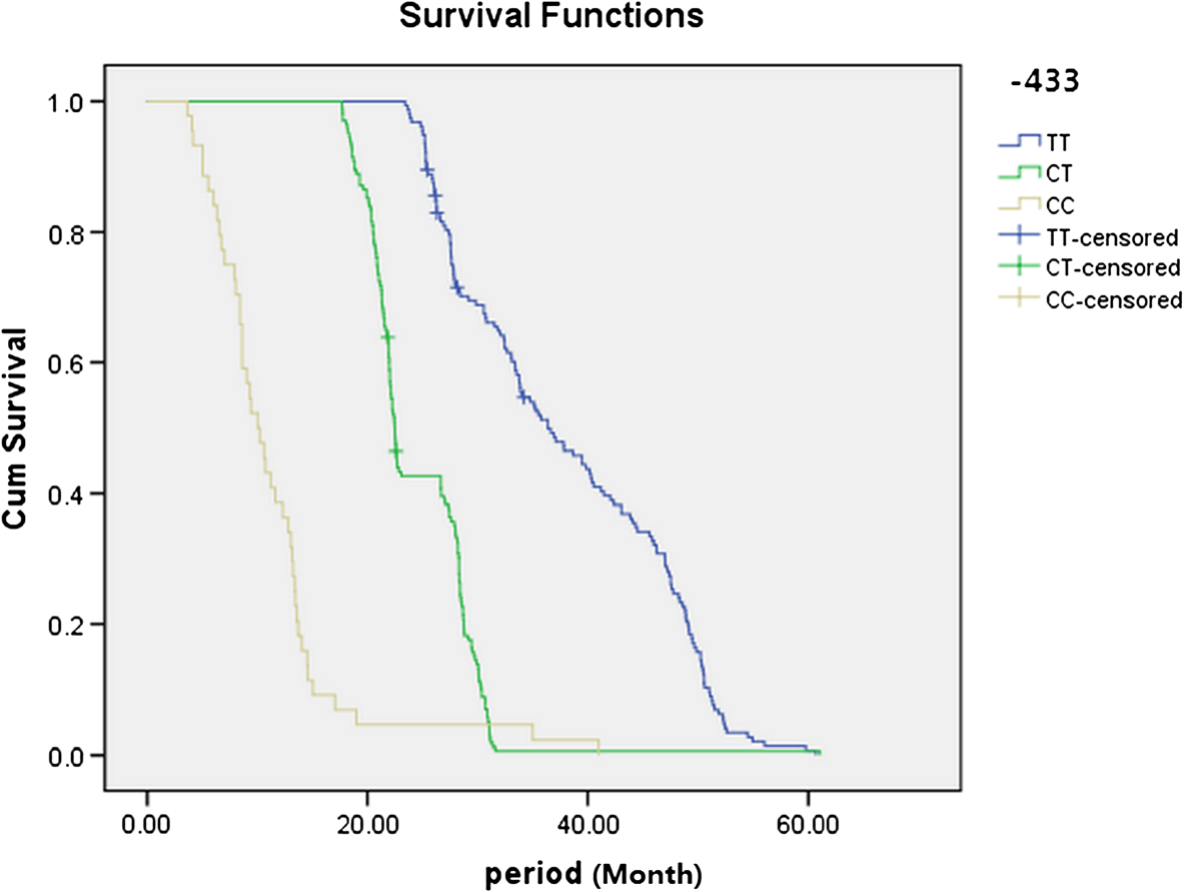
Kaplan-Meier survival is significantly lower in lung cancer patients with the C/C genotype as compared to the other two genotypes at nt −443 in OPN promoter.

Kaplan-Meier estimates of different genotypes at nt −443 in the OPN promoter were shown in Figure 1. The survival rates for patients with the C/C genotype were significantly lower than the survival rates for patients with the other two genotypes (C/T, T/T), and C/T genotype was also significantly lower than the survival rates for patients with T/T genotype. There were no significant associations between survival and genotypes at the other sites (nt −156 and nt −66, data not shown).

## Discussion

Based on my knowledge, it is first time to report the relationship between OPN polymorphisms and bone metastasis among NSCLC patients. Lots of evidence suggests that OPN plays a role in the regulation of tumor metastasis and that OPN expression is particularly high in metastatic tumors [20-22]. OPN is overexpressed in cancers that have a high propensity for forming bone metastases. In bone metastases, OPN is generally associated with the interface between the carcinoma and the bone surface, and this appears to be related to increased bone resorptive activity by osteoclasts [23]. Moreover, high OPN expression in the primary tumor is associated with early metastasis and poor clinical outcome in human gastric cancer and other cancers [19,20,24-27].

A recent study suggested that the OPN promoter was associated with NSCLC [28]. In the present study, we focused on the association of these SNPs with TNM stages of lung cancer, especially for bone metastasis. Although the distribution of genotypes in the OPN promoter was not significantly different between lung cancer patients and healthy controls, there were significant differences in the distribution of genotypes (CC) at nt −443 between patients with stage IV and other stage lung cancer (Table 4). The survival rates for patients with the C/C genotype were significantly lower than the survival rates of the other two genotypes (C/T, T/T; Figure 1).

Recent study proved that the haplotype -443C/-156 G/-66 T is associated with significantly enhanced promoter activity compared to five other allelic variants tested [29]. A recent study on melanoma metastases found that those homozygous for the -443C allele expressed significantly higher levels of OPN mRNA compared to those that were either heterozygous (CT) or homozygous for the −443 T allele [30]. Transcription factor c-Myb binds to the region of the OPN promoter in an allele-specific manner and induces enhanced activity of the -443C compared to the −443 T OPN promoter [31]. Taken together, these data suggest that the variation at nt −443 in the OPN promoter plays a role in GC progression and metastasis, especially for the CC genotype at nt −443 in the OPN promoter. Whether the polymorphisms of OPN are related to expression of OPN in cancer patients remain unknown. Over-expression of OPN was found in lung cancer samples in a previous study [16], and the alteration of the −443 T → C promoter region could significantly increase the promoter activity by Dual Luciferase Reporter Assay System [19].

In the present study, we found that the CT genotype at nt −443 in the OPN promoter showed significant differences between stages III + IV and stage I + II lung cancer, but no significant difference between stage IV and sum of other stages of lung cancer (Table 4); and for the CC genotype, there was significant difference between stage IV and other single stages or combination of any other stages. The main reason for this may be due to the limited number of patients in CC type subgroups. It is also possible that the CC genotype has more enhanced transcription activity of the region of the OPN promoter compared to CT genotypes [30]. Among total 31 CC genotype patients, 20 patients were diagnosed as bone metastasis, it is extremely high, but there is no significant difference on the ratio of CC type between lung cancer patients and healthy controls. The main reason for this, we hypothesize that OPN is a not key factor for initiating lung cancer, but once the carcinogenesis occurred, OPN will enhance this process effectively, especially for distant metastasis and bone metastasis, which is consistent with previous study. However, the further study is needed to investigate this hypothesis.

There are also some drawbacks in the present study, one of them is because all the subjects are Chinese individuals, the results should be interpreted with caution and need to be confirmed in larger and ethnically divergent population samples. On the other hand, the number of stage IV patients without bone metastasis in the current study is not high enough, so the large-population research is needed to make stronger conclusion about the association between bone metastasis formation and −433 polymorphisms.

## Conclusions

In summary, -443C/T of OPN is a potential biomarker for predicting prognosis of lung cancer, especially for bone metastasis.

## Competing interests

The authors declare that they have no competing interests.

## Authors’ contributions

YZC and JML defined the research theme. YZC and HCL designed methods and experiments, carried out the laboratory experiments, analyzed the data. WLW and YL co-worked on associated data collection and their interpretation. All authors read and approved the final manuscript.

## Supplementary Material

Additional file 1: Figure S1 Schematic diagram and sequencing data of the OPN promoter. Representative figure for the sequencing analysis on the promoter. The SNP nt −443 has the following alleles: CC, CT, and TT. There is a small insertion at nt-156, which has three alleles: G/G, G/GG, GG/GG. The SNP nt −66 has only one allele: TT. Click here for file

## References

[B1] Shen H Li Y Liao Y Zhang T Liu Q Du J Lower blood calcium associates with unfavorable prognosis and predicts for bone metastasis in NSCLC PLoS One 2012 7 e34264 10.1371/journal.pone.0034264 22479582 PMC3316630

[B2] Bi N Yang M Zhang L Chen X Ji W Ou G Lin D Wang L Cyclooxygenase-2 genetic variants are associated with survival in unresectable locally advanced non-small cell lung cancer Clin Canc Res: an official journal of the American Association for Cancer Research 2010 16 2383 2390 10.1158/1078-0432.CCR-09-2793 20332326

[B3] Gandara D Narayan S Lara PN Jr Goldberg Z Davies A Lau DH Mack P Gumerlock P Vijayakumar S Integration of novel therapeutics into combined modality therapy of locally advanced non-small cell lung cancer Clin Canc Res: an official journal of the American Association for Cancer Research 2005 11 5057s 5062s 10.1158/1078-0432.CCR-05-9012 16000614

[B4] Lee CB Stinchcombe TE Rosenman JG Socinski MA Therapeutic advances in local-regional therapy for stage III non-small-cell lung cancer: evolving role of dose-escalated conformal (3-dimensional) radiation therapy Clin Lung Canc 2006 8 195 202 10.3816/CLC.2006.n.047 17239295

[B5] Liu SK Olive PL Bristow RG Biomarkers for DNA DSB inhibitors and radiotherapy clinical trials Cancer Metastasis Rev 2008 27 445 458 10.1007/s10555-008-9137-8 18516501

[B6] Hashisako M Wakamatsu K Ikegame S Kumazoe H Nagata N Kajiki A Flare phenomenon following gefitinib treatment of lung adenocarcinoma with bone metastasis Tohoku J Exp Med 2012 228 163 168 10.1620/tjem.228.163 23036980

[B7] Pathi SP Kowalczewski C Tadipatri R Fischbach C A novel 3-D mineralized tumor model to study breast cancer bone metastasis PLoS One 2010 5 e8849 10.1371/journal.pone.0008849 20107512 PMC2809751

[B8] Santini D Schiavon G Vincenzi B Gaeta L Pantano F Russo A Ortega C Porta C Galluzzo S Armento G Receptor activator of NF-kB (RANK) expression in primary tumors associates with bone metastasis occurrence in breast cancer patients PLoS One 2011 6 e19234 10.1371/journal.pone.0019234 21559440 PMC3084800

[B9] Coleman RE Clinical features of metastatic bone disease and risk of skeletal morbidity Clin Canc Res: an official journal of the American Association for Cancer Research 2006 12 6243s 6249s 10.1158/1078-0432.CCR-06-0931 17062708

[B10] Clezardin P Teti A Bone metastasis: pathogenesis and therapeutic implications Clin Exp Metastasis 2007 24 599 608 10.1007/s10585-007-9112-8 18008175

[B11] Vetrone SA Montecino-Rodriguez E Kudryashova E Kramerova I Hoffman EP Liu SD Miceli MC Spencer MJ Osteopontin promotes fibrosis in dystrophic mouse muscle by modulating immune cell subsets and intramuscular TGF-beta J Clin Invest 2009 119 1583 1594 10.1172/JCI37662 19451692 PMC2689112

[B12] Huan JL Xing L Qin XJ Gao ZG Pan XF Zhao ZD Expression and clinical significance of osteopontin in calcified breast tissue Asian Pac J Cancer Prev 2012 13 5219 5223 10.7314/APJCP.2012.13.10.5219 23244138

[B13] Senger DR Perruzzi CA Secreted phosphoprotein markers for neoplastic transformation of human epithelial and fibroblastic cells Cancer Res 1985 45 5818 5823 4053052

[B14] Uaesoontrachoon K Yoo HJ Tudor EM Pike RN Mackie EJ Pagel CN Osteopontin and skeletal muscle myoblasts: association with muscle regeneration and regulation of myoblast function in vitro Int J Biochem Cell Biol 2008 40 2303 2314 10.1016/j.biocel.2008.03.020 18490187

[B15] Staal A van Wijnen AJ Birkenhager JC Pols HA Prahl J DeLuca H Gaub MP Lian JB Stein GS van Leeuwen JP Stein JL Distinct conformations of vitamin D receptor/retinoid X receptor-alpha heterodimers are specified by dinucleotide differences in the vitamin D-responsive elements of the osteocalcin and osteopontin genes Mol Endocrinol 1996 10 1444 1456 10.1210/me.10.11.1444 8923469

[B16] Jin Y Tong DY Tang LY Chen JN Zhou J Feng ZY Shao CK Expressions of Osteopontin (OPN), alphanubeta3 and Pim-1 Associated with Poor Prognosis in Non-small Cell Lung Cancer (NSCLC) Chin J Cancer Res 2012 24 103 108 10.1007/s11670-012-0103-1 23359766 PMC3555265

[B17] Chung JH Park MS Kim YS Chang J Kim JH Kim SK Usefulness of bone metabolic markers in the diagnosis of bone metastasis from lung cancer Yonsei Med J 2005 46 388 393 10.3349/ymj.2005.46.3.388 15988811 PMC2815816

[B18] Aruga A Koizumi M Hotta R Takahashi S Ogata E Usefulness of bone metabolic markers in the diagnosis and follow-up of bone metastasis from lung cancer Br J Cancer 1997 76 760 764 10.1038/bjc.1997.458 9310242 PMC2228042

[B19] Zhao F Chen X Meng T Hao B Zhang Z Zhang G Genetic polymorphisms in the osteopontin promoter increases the risk of distance metastasis and death in Chinese patients with gastric cancer BMC Cancer 2012 12 477 10.1186/1471-2407-12-477 23072570 PMC3517443

[B20] Chiu YW Tu HF Wang IK Wu CH Chang KW Liu TY Kao SY The implication of osteopontin (OPN) expression and genetic polymorphisms of OPN promoter in oral carcinogenesis Oral Oncol 2010 46 302 306 10.1016/j.oraloncology.2010.01.018 20219412

[B21] Rodrigues LR Teixeira JA Schmitt FL Paulsson M Lindmark-Mansson H The role of osteopontin in tumor progression and metastasis in breast cancer Canc Epidemiol Biomarkers Prev 2007 16 1087 1097 10.1158/1055-9965.EPI-06-1008 17548669

[B22] Wai PY Kuo PC The role of osteopontin in tumor metastasis J Surg Res 2004 121 228 241 10.1016/j.jss.2004.03.028 15501463

[B23] Bourguignon LY Zhu H Shao L Zhu D Chen YW Rho-kinase (ROK) promotes CD44v(3,8-10)-ankyrin interaction and tumor cell migration in metastatic breast cancer cells Cell Motil Cytoskeleton 1999 43 269 287 10.1002/(SICI)1097-0169(1999)43:4<269::AID-CM1>3.0.CO;2-5 10423269

[B24] Chu M Yang P Hu R Hou S Li F Chen Y Kijlstra A Elevated serum osteopontin levels and genetic polymorphisms of osteopontin are associated with Vogt-Koyanagi-Harada disease Invest Ophthalmol Vis Sci 2011 52 7084 7089 10.1167/iovs.11-7539 21810982

[B25] Alain K Karrow NA Thibault C St-Pierre J Lessard M Bissonnette N Osteopontin: an early innate immune marker of Escherichia coli mastitis harbors genetic polymorphisms with possible links with resistance to mastitis BMC Genom 2009 10 444 10.1186/1471-2164-10-444 PMC2761946 19765294

[B26] Niino M Kikuchi S Fukazawa T Yabe I Tashiro K Genetic polymorphisms of osteopontin in association with multiple sclerosis in Japanese patients J Neuroimmunol 2003 136 125 129 10.1016/S0165-5728(03)00004-3 12620651

[B27] Wu CY Wu MS Chiang EP Wu CC Chen YJ Chen CJ Chi NH Chen GH Lin JT Elevated plasma osteopontin associated with gastric cancer development, invasion and survival Gut 2007 56 782 789 10.1136/gut.2006.109868 17148500 PMC1954839

[B28] Chang YS Kim HJ Chang J Ahn CM Kim SK Elevated circulating level of osteopontin is associated with advanced disease state of non-small cell lung cancer Lung Canc 2007 57 373 380 10.1016/j.lungcan.2007.04.005 17513004

[B29] Brown LF Papadopoulos-Sergiou A Berse B Manseau EJ Tognazzi K Perruzzi CA Dvorak HF Senger DR Osteopontin expression and distribution in human carcinomas Am J Pathol 1994 145 610 623 8080043 PMC1890312

[B30] Schultz J Lorenz P Ibrahim SM Kundt G Gross G Kunz M The functional -443T/C osteopontin promoter polymorphism influences osteopontin gene expression in melanoma cells via binding of c-Myb transcription factor Mol Carcinog 2009 48 14 23 10.1002/mc.20452 18459127

[B31] Iwasaki H Shinohara Y Ezura Y Ishida R Kodaira M Kajita M Nakajima T Shiba T Emi M Thirteen single-nucleotide polymorphisms in the human osteopontin gene identified by sequencing of the entire gene in Japanese individuals J Hum Genet 2001 46 544 546 10.1007/s100380170037 11558904

