# Supplementary figures and images for: Osteopontin genetic variants are associated with overall survival in advanced non-small-cell lung cancer patients and bone metastasis
Source: J Exp Clin Cancer Res. 2013 Jul 24;32(1):45. doi: 10.1186/1756-9966-32-45 (PMC3728114; doi:10.1186/1756-9966-32-45)

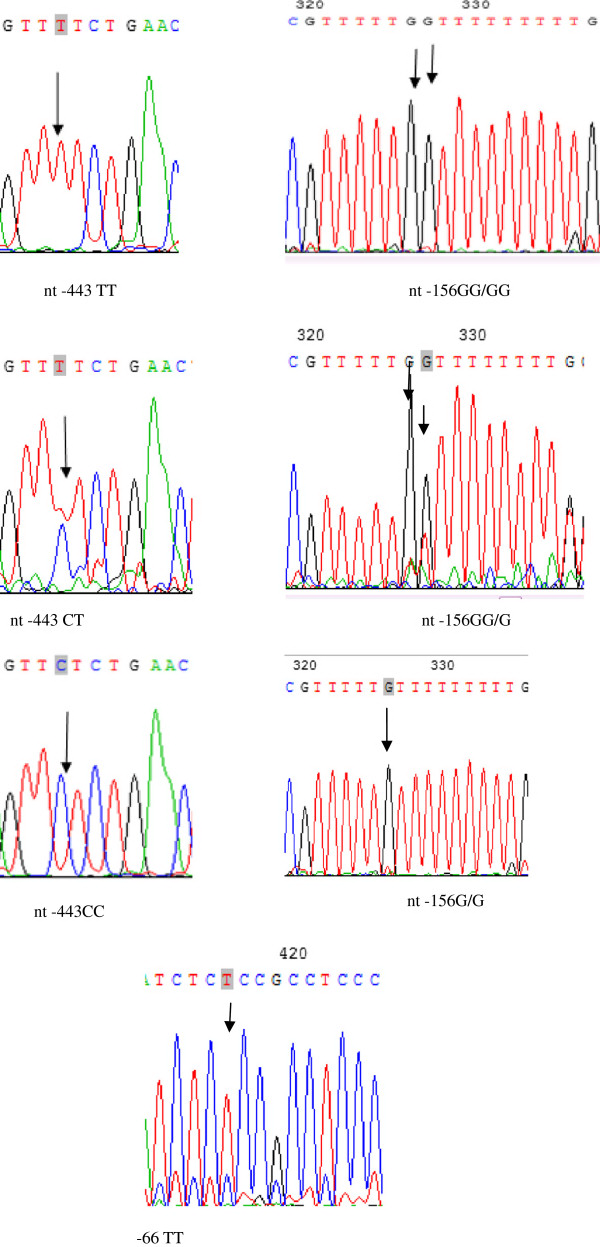

Supplement: Additional file 1: Figure S1 — Schematic diagram and sequencing data of the OPN promoter. Representative figure for the sequencing analysis on the promoter. The SNP nt −443 has the following alleles: CC, CT, and TT. There is a small insertion at nt-156, which has three alleles: G/G, G/GG, GG/GG. The SNP nt −66 has only one allele: TT. [file 1756-9966-32-45-S1.tiff]
